# The role of the community in providing psychological and social support after catastrophic events

**DOI:** 10.1192/j.eurpsy.2024.982

**Published:** 2024-08-27

**Authors:** L. Mužinić Marinić, I. Marinić

**Affiliations:** Clinic for Psychiatry, University Hospital Dubrava, Zagreb, Croatia

## Abstract

**Introduction:**

Natural disasters are a risk for significantly disrupting the quality of life as a result of changes in life circumstances they bring, such as endangering health, property, existential issues, and can lead to social exclusion. They can also affect mental health and increase the risk of developing psychiatric disorders.

**Objectives:**

To show the impact of natural disasters on the psychosocial functioning of people in the affected area and the importance of adequate preparedness of the social community, including mobile teams, with an emphasis on providing somatic, psychological, and social support.

**Methods:**

Data were collected from research on the consequences of major natural disasters and providing psychological, psychiatric and social support to the affected population.

**Results:**

After natural disasters, there are significant changes in social functioning with the possible development of mental health problems. It is especially evident in sudden and intense catastrophic events.

**Conclusions:**

In addition to the immediate provision of psychiatric and psychological assistance to victims, people who have experienced a catastrophic event need to be provided with immediate and continuous assistance and socioeconomic support, due to the need for better social inclusion and return to their role in the community.

**Disclosure of Interest:**

None Declared

